# Oxygen Availability during Growth Modulates the Phytochemical Profile and the Chemo-Protective Properties of Spinach Juice

**DOI:** 10.3390/biom9020053

**Published:** 2019-02-04

**Authors:** Francesco Milano, Francesca Mussi, Silvia Fornaciari, Meltem Altunoz, Luca Forti, Laura Arru, Annamaria Buschini

**Affiliations:** 1 Dipartimento di Scienze Chimiche, della Vita e della Sostenibilità Ambientale, Università degli Studi di Parma, Parco Area delle Scienze 11/A, 43124 Parma, Italy; francesco.milano@gmail.com (F.M.); francesca.mussi@unipr.it (F.M.); annamaria.buschini@unipr.it (A.B.); 2 Dipartimento di Scienze della Vita, Università degli Studi di Modena e Reggio Emilia, via Amendola 2, 42122 Reggio Emilia, Italy; fornaciari04@gmail.com (S.F.); meltem.altunoz@unimore.it (M.A.); luca.forti@unimore.it (L.F.); 3 COMT (Centro di Oncologia Molecolare e Traslazionale), Università degli Studi di Parma, Parco Area delle Scienze 11/A, 43124 Parma, Italy

**Keywords:** *Spinacia oleracea* L., antioxidant activity, anti-proliferative activity, Comet Assay, Liquid Chromatography-Mass Spectrometry, HT29 cell line

## Abstract

Fruits and vegetables are a good source of potentially biologically active compounds. Their regular consumption in the human diet can help reduce the risk of developing chronic diseases such as cardiovascular diseases and cancer. Plants produce additional chemical substances when subject to abiotic stress or infected by microorganisms. The phytochemical profile of spinach leaves (*Spinacia oleracea* L.), which is a vegetable with widely recognized health-promoting activity, has been affected by applying root hypoxic and re-oxygenation stress during plant growth. Leaf juice at different sampling times has been subject to liquid chromatography mass spectrometry (LC-MSn) analysis and tested on the human colorectal adenocarcinoma cell line HT29 by using the Comet assay. The cells were previously treated with H_2_O_2_ to simulate the presence of an oxidative stress (as in colon cancer condition) and the leaf juice application resulted in a significant antioxidant and protective in vitro effect. The duration of the hypoxic/re-oxygenation stress imposed on the plant reflects the antioxidant leaf juice content. After hypoxic stress (24 h) and reoxygenation (2 h), we show a decrease (50%) of the relative abundance of the principal identified antioxidant molecules but a higher antioxidant activity of the spinach juice on HT29 cells (20%). Data shows a complex relation between plant growing conditions and the modulation of secondary metabolites content in leaf juice that results in different chemo-protective activities in colon cancer cells.

## 1. Introduction

A diet rich in fruits and vegetables has beneficial preventive effects on human health, which helps the free-radical scavenging and the metal ions chelating mechanisms of the human body due to the presence of natural occurring antioxidant compounds such as polyphenols. The importance of these scavenging mechanisms lies in reducing the harmful effect of the oxidative species on DNA, proteins, and lipids at a cellular level [1,2]. Similar to many other secondary metabolites, the antioxidant species can reach major or minor concentrations inside the plant based on the plant’s surrounding conditions [3,4]. An example of these conditions, which can affect the secondary metabolites’ presence, is the variation in root oxygen availability [5,6,7,8]. The plant carries out an integrated molecular, metabolic, and physiological response in order to survive to the sub-optimal oxygen condition and the presence of antioxidant compounds plays a key role in tolerating the related oxidative stress damage [9,10,11,12,13,14].

In vitro experiments on human cell lines demonstrate that plant antioxidants’ application can lead to an anti-proliferative activity related to a set of epigenetic alterations, which involves signal transduction pathways [15,16]. On the other hand, the administration of a phyto-complex rich in antioxidant species can lead to a pro-oxidant activity [17,18]. In this case, the hypothesis is that the already unbalanced redox state (with a high amount of Reactive Oxygen Species (ROS) typical of cancer cells) makes the cells more susceptible to any additional oxidative stress [18].

*Spinacia oleracea* L. is a well-known herbaceous plant belonging to the family of Chenopodiaceae, which comes from Central and Southwestern Asia. According to the Agricultural Research Service of the U.S. Department of Agriculture, 100 g of fresh spinach provide approximately 20% or more of the recommended dietary intake of β-carotene (provitamin A), lutein, folate (vitamin B9), α-tocopherol (vitamin E), and ascorbic acid (vitamin C). Moreover, spinach leaves contain flavonoids [19] and phenolic acids such as ferulic acid, *orto*-coumaric, and *para*-coumaric acids [20].

In 2009, Hait-Darshan et al. [21] isolated from spinach leaves a mixture of antioxidants defined NAO (Natural Anti-Oxidant) containing aromatic polyphenols and derivatives of the glucuronic acid. NAO can counteract free radicals [22,23] that show anti-proliferative and anti-inflammatory potential both in vivo and in vitro [24]. Spinach leaves reveal an important anti-tumor function in various types of cancers, including lung, prostate, breast, colon, and ovarian cancers [22,25]. Spinach is also rich in ecdysteroids, namely 20-hydroxyecdysone (20HE). Phytoecdysteroids are structurally similar or identical to the hormones involved in the processes of molting and metamorphosis in insects. They induce a wide range of effects in mammals, including the increase of physical performance without training [26].

In this study spinach plants (*S. oleracea* L.), which hold an already well-known source of bioactive molecules and healthy compounds [19,27,28], have been subject to hypoxia and subsequent re-oxygenation. Spinach leaf juice was then tested on the human HT29 colon cancer cell line and the relationship between the modulation in the phytochemical composition and the response of the human cells has been investigated.

## 2. Materials and Methods 

### 2.1. Reagents

Salts, hexane, acetone, acetic acid, potassium peroxydisulfate, and ascorbic acid were purchased from Sigma–Aldrich (Milan, Italy). Dulbecco’s modified Eagle medium (DMEM), penicillin, streptomycin, L-glutamine, fetal bovine serum, trypsin, and EDTA were purchased from Lonza (Vievers, Belgium).

### 2.2. Plant Material

*Spinacia oleracea* L. (cv. Parrot) seeds were sterilized in a 2% (*w*/*v*) solution of sodium hypochlorite for 15 min, well rinsed in distilled water, and germinated on sterile sand in a growth chamber at 24 °C. After 3 days from germination, uniform seedlings were transferred into a hydroponic floating system (three tanks with 40 plants each filled with 10 L of a modified version of Hoagland solution as growth medium) [16]. Then it was maintained in a normoxic condition (Boyu S-1000 air pump: 4.2 L/min, pressure 0.014 MPa, power 3 W) while constantly monitoring the parameters, according to what was found in the literature [29,30]. The hydroponic culture system was set in a growth chamber under 40% to 60% relative humidity and 24 °C/19 °C day/night temperature.

After 30 days from starting germination, at the stage of 5–6 leaves per plant, a subset of plants (two tanks) were exposed to the hypoxic condition by suspending aeration and covering the surface of the tank to prevent gas exchange with the atmosphere. Oxygen concentration, pH, and a temperature of the nutrient solution were continuously monitored throughout the experiment by means of a portable dissolved oxygen meter (Oxy-Check HI 9147, Hanna Instruments) (Supplementary Materials Table S1). Hypoxia (O_2_ < 0,4 mg/L), which was reached about 8 to 12 h after aeration suspension. After 24 h, one tank was reaerated. Reaeration caused a quick rise in O_2_ concentration, which brings back oxygen levels to a value of 6 mg/L within 2 h. The experiment was repeated three times and each time the leaves were sampled in triplicate at the following timings: 0 h, 24 h, and 26 h (2 h after re-oxygenation). It was then stored at –80 °C until use.

### 2.3. Spinach Juice

Spinach leaves were grounded in mortar with liquid nitrogen in excess. The resulting juice was weighted and transferred into a 10 mL syringe and then filtered through a Nylon filter (60 µm). The juice was centrifuged in falcon at 10,000 rpm for 10 minutes at 4 °C. The supernatant filtered with a 0.22 µm filter was then stored at –20 °C.

### 2.4. Liquid Cromatography-Mass Spectrometry Analysis

Spinach juice was dried using the Speed Vacuum Concentrator Eppendorf 5301 (Eppendorf, AG, Hamburg, Germany), solved back with 500 µL of a 1:9 (*v*/*v*) water: acetone solution, and then diluted 1:10 into a solution of acetonitrile/water 1:9 (*v*/*v*). Mass spectral data were acquired on the 6310A Ion Trap Liquid Chromatography Mass Spectrometry (LC-MS), and designed with a Quadrupole ion trap mass spectrometry to detect the exact mass of the eluted molecules. Chromatography was performed on an Agilent 1200 LC system (Agilent Technologies, Palo Alto, CA, USA) equipped with an ZORBAX SB-C18 column (21 × 30 mm, 35 µm particle size). (Supplementary Materials Figures S1 and S2).

### 2.5. Trypan Blue Exclusion Cell Viability Assay

HT29 cells, kindly obtained from the Northern Ireland Center for Food and Health, were seeded in six well plates (2 mL/well) at the density of 2 × 10^5^ cell/well. Cells were treated with 10%, 25%, and 50% of spinach juice for 24 h and then counted. Each experiment was performed in triplicate. For cell counting, after 24 h of treatment, the cells were trypsinized and re-suspended in DMEM supplemented with 1% glutamine, 1% penicillin/streptomycin, and 10% fetal bovine serum (all purchased from Lonza, Vievers, Belgium) with a sterile 1 mL pipette. In addition, 30 µL of cell suspension were mixed with the same amount of trypan blue (Trypan Blue Stain 0.4%—Lonza, Walkersville, MD 21793 USA). Cells were visually examined at 400× magnification to determine whether they took up or excluded dye. A viable cell presents a clear cytoplasm whereas a nonviable cell shows a blue cytoplasm. For each sample, 100 cells were scored.

### 2.6. Comet Test

Single cell gel electrophoresis (Comet Assay) was used to assess genotoxicity and antioxidant activity [16,31]. The HT29 cells were seeded at the concentration of 1 × 10^5^/mL in six well plates in DMEM supplemented with 1% glutamine, 1% penicillin/streptomycin, and 10% fetal bovine serum. After 24 h from the seeding, the HT29 cells were treated with spinach extract or with different concentrations of vitamin C for the genotoxicity, using non-toxic concentrations, as detected by the trypan blue cell viability assay. To obtain the different juice solutions, aliquots of juice were diluted in DMEM supplemented with 1% glutamine, 1% penicillin/streptomycin, and 10% fetal bovine serum. After 24 h of treatment, the cells were trypsinized and re-suspended again into supplemented DMEM at a concentration of 5 × 10^4^ cell/mL to prepare the slides for the Comet Assay.

To assay the antioxidant activity, the same procedure was followed, except for the use of an oxidative-inducing agent (H_2_O_2_ 100 µM, 5 min) on the cells pre-treated with spinach extract or with vitamin C. At least three independent experiments were performed with each extract (see Supplementary Materials Figure S3 for an example of HT29 cells nuclei under different treatments).

Slides were examined at 400× magnification under a Leica DMLS fluorescence microscope (excitation filter BP 515–560 nm, barrier filter LP 580 nm), using an automatic image analysis system (Comet Assay III—Perceptive Instruments Ltd., Bury St Edmunds, UK).

## 3. Results and Discussion

### 3.1. Oxygen Availability Effect on Spinach Leaf Juice Composition

Juice from spinach leaves sampled after 24 h from the beginning of the hypoxic stress induction (T24) and after 2 h from the subsequent re-oxygenation (T26) has been analyzed by means of HPLC coupled to MSn. LC-MSn obtained data have been compared with spinach MS data provided in literature to help identify the most significant antioxidant secondary metabolites. Table 1 shows the major peaks and their relative abundance referred to the relative normoxic control (i.e., T24 hypoxic/T24 normoxic, T26 reoxigenated/T26 normoxic) based on the integrated area of the peak. 

As expected, and according to the literature, the presence or abundance of many low-molecular weight antioxidants (such as ascorbic acid, ferulic acid, and α-tocopherol) is influenced by stress conditions. Secondary metabolites with antioxidant properties have already been shown to modulate their abundance in response to an oxidative stress such as hypoxia [37], which is a condition under which a high generation of ROS can be expected [38]. The same applies to re-oxygenation, where a considerable formation of ROS and free radicals occurs within a few minutes after the restoration of the oxygen supply [39]. 

In this experiment, the presence of significant antioxidant species increases after 24 h from the beginning of the plant hypoxic stress (T24) and it lowers back during re-oxygenation (T26). In fact, generation of ROS (especially hydrogen peroxide -H_2_O_2_- and superoxide -O_2_-) is characteristic of hypoxic stress and it is enhanced during re-oxygenation [13]. Thus, the antioxidant system (including low molecular mass antioxidants) helps prevent cellular components (e.g. membranes, proteins, nucleic acids) damage especially during oxygen reintroduction [40]. Specific glucuronic acid derivatives and jaceidin, patuletin, and spinacetin derivatives of flavonoids have already been found in spinach [41]. Some of these typical flavonoids have been confirmed as major peaks in this study. In addition, an increase in the phenolic antioxidant content of the leaf juice from hypoxic plants has been registered. This increase could be related to the evidence that some flavonoids are good iron-ions chelators, as suggested by Leopoldini and colleagues [42]. This behavior interferes with the generation of ROS inhibiting the oxidative reactions via the Fenton reaction. An enhanced production of antioxidants during hypoxia represents both a way to inhibit the generation of ROS itself and a preventive action against ROS generation during the subsequent re-oxygenation. Re-oxygenation seems to wear most of these antioxidants out, which lowers their amount within two hours from the reaeration of the growing solution. 

The presence of the phytoecdysteroid 20-hydroxyecdysone has also been detected. It is another well-known spinach secondary metabolite whose healthy properties have been widely proven [43,44,45]. Even if the 20-hydroxyecdysone does not belong to the group of common antioxidants carrying on a ROS defense role in the case of plant hypoxia, in this study, its abundance has shown to be modulated in response to the stress condition.

### 3.2. Effect on Viability and Antioxidant Activity on HT29 Human Colon Cancer Cell Line

Spinach leaf juice from plants under normoxia, hypoxia, and re-oxygenated state has been tested on HT29 human colon cancer cells in order to evaluate the possible anti-proliferative effects. For each condition, three different juice concentrations (10%, 25%, and 50%) have been administered to the cancer cells (Table 2).

With regards to cell viability, the leaf juice from plants under normoxic and hypoxic conditions at the highest concentration tested (50%) induces a high mortality rate (>30%), which is the highest for the hypoxic condition (>70%). For all the juices (from normoxic, hypoxic, and reoxigenated plants), the lowest concentration tested (10%) does not influence cell viability and, thus, this concentration has been chosen as the highest threshold value to be tested in further assays. 

**Figure 1:**
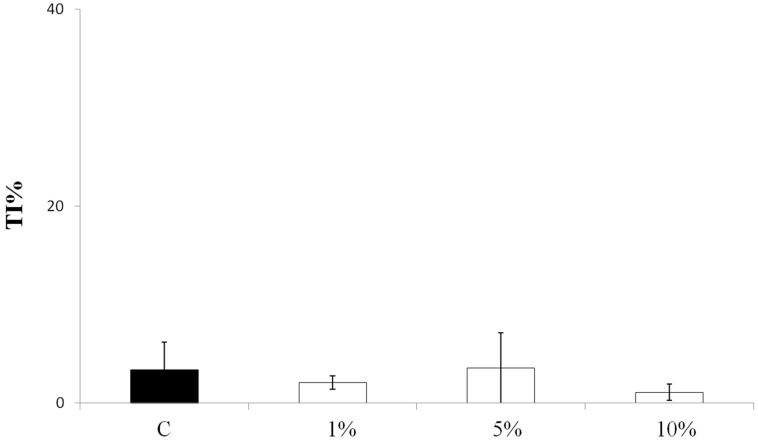
Genotoxic activity of spinach juice from leaves of plants grown in a normoxic condition. The activity was evaluated testing 3 different leaf juice concentrations (1%, 5%, and 10%) by Comet assay on HT29 cells after 24 h of treatment. Each histogram is the result of the mean ± SD of three determinations. TI%: tail intensity percentage. C: untreated control cells. SD: standard deviation.

A Comet assay with three different leaf juice concentrations (1%, 5%, and 10%) from normoxic spinach plants was carried out to test if the spinach juice could have a genotoxic effect on HT29 human colon cancer cells. The results did not show any increase in DNA migration, which suggests that the leaf juice has no genotoxic effect at any of the concentrations tested (Figure 1).

**Figure 2:**
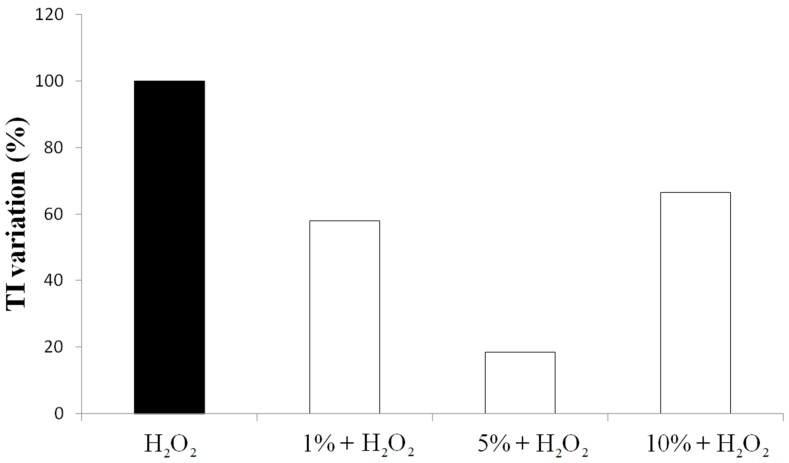
Antioxidant activity of spinach juice on HT29 colon cancer cells. Antioxidant activity has been evaluated by Comet assay on HT29 cells after 24 h of treatment with spinach juice from normoxic plants at different concentrations (1%, 5%, and 10%) and 5 min of H_2_O_2_ (100 µM) stress. On the *y*-axis, the Tail Intensity (TI) variation (%): (TI _sample_ + H_2_O_2_/TI H_2_O_2_) x 100 is reported.

Spinach leaf juice has also been administrated to HT29 cells after preventive treatment with H_2_O_2_ as an oxidizing agent. The juice has been tested at three different concentrations (Figure 2). Independently from the concentration, the juice shows an antioxidant activity with an evident reduction of the H_2_O_2_ oxidative effect on DNA.

In particular, the 1% juice solution shows a reduction of 40% of DNA migration. The 5% juice shows the highest antioxidant activity with a reduction of the 80% of DNA damage. The 10% juice decreases the DNA migration of 33% with respect to the H_2_O_2_ alone. This is another example that the antioxidant activity of a chemical compound is not always dose-dependent. In the reported experiment, the best antioxidant effect is recorded when the 5% juice solution is administered to HT29 cells. The observed hormetic behavior resembles the response of cancer cell lines to vitamin C and plant flavonoids, which confirms the evidence reported in literature where an excess of exogenous antioxidants can alter the endogenous antioxidant balance and affects, in turn, the defense response [46,47,48]. Thus, only the 1% and 5% juice concentrations (which showed a marked antioxidant effect) have been chosen for further experiments. 

**Figure 3:**
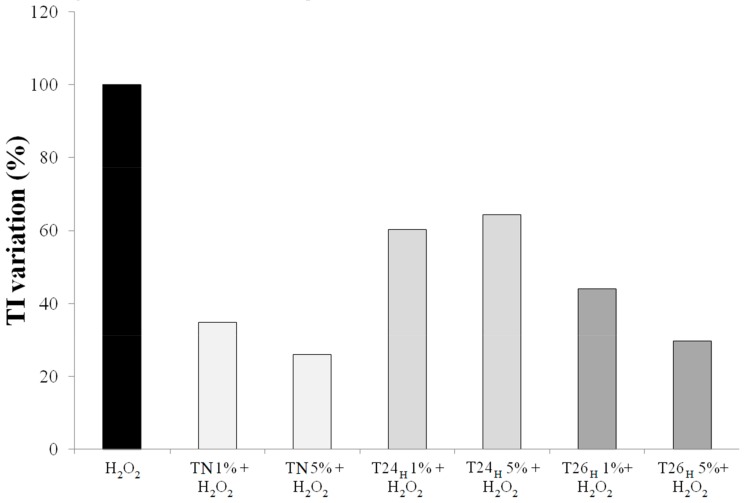
Antioxidant activity of spinach juice evaluated by the Comet assay on HT29 cells treated (24 h) with normoxic (T_N_) and hypoxic spinach juice (T24_H_, T26_H_) at different concentrations (1% and 5%) and stressed with H_2_O_2_ (100 µM) for 5 min. In the *y* axis, the Tail Intensity (TI) variation (%): (TI_sample + H_2_O_2__/TI_H_2_O_2__) × 100 is reported.

The antioxidant effect of spinach leaf juice from plants subject to 24 h of hypoxia (T24Hy) on HT29 cells was not comparable to the one observed with the administration of leaf juice from normoxic plants (Figure 3). This is coherent with the fact that a higher antioxidant concentration in the juice (as verified with the LC-MSn analysis) can induce a lower antioxidant activity on human cells. It happens that, when the antioxidants concentration exceeds a threshold over which the more antioxidant species there are, the less antioxidant effect there is. Leaf juice from hypoxic plants after 2 h from re-oxygenation did not add any antioxidant activity to the results obtained by administration of leaf juice from normoxic plants sampled at the same time. This is due to the content of antioxidant compounds being diminished to the normoxic plant level.

## 4. Conclusions

Spinach leaves contain water-soluble natural and powerful compounds with potential antioxidant activity. The plant can modulate the leaf content of these chemical compounds, according to the stress the plant has to withstand. LC-MSn analysis evidence in the spinach leaf juice, the presence of both common and more peculiar flavonoid compounds, flavonol derived molecules, and low molecular mass antioxidants appear to be modulated when plants are subject to hypoxic stress. Tested on the HT29 colon tumor cell line previously treated with H_2_O_2_, spinach leaf juice shows both anti-proliferative and antioxidant effects. The anti-proliferative effect (but not the antioxidant one) is markedly enhanced when the juice comes from the leaves of plants treated for 24 h under hypoxic stress. This behavior suggests a possible pro-oxidant activity of the hypoxic leaf extract on HT29 cells: many phytochemicals, under certain circumstances or over a determined threshold, exert a different effect and become pro-oxidant when acting on altered systems (such as vitamin C).

The balance between antioxidant and pro-oxidant activity of a chemical on a cell line model depends on a specific set of conditions such as on the stages of carcinogenesis and on the physiological concentration of ROS [49]. It is essentially an integrated approach to analyze the data and to understand when and to what extent the antioxidant or pro-oxidant effects are promoted. In this context, the results reported here require a deeper examination to relate the leaf juice composition with the effects in human cancer cells. It would also be interesting to unravel the molecular response pathways involved and to check the effect of spinach leaf juice on normal cells or other cells. This could help improve understanding if the observed cell response is related to a specific cell tissue or if it can be extended to other tumor cell lines.

## Figures and Tables

**Table 1:** Liquid chromatography-mass spectrometry analysis: principal identified peaks and relative abundance ^a^.

Peak Nr.	Ret Time (min)	[M-H]^−^ *m/z*	[M+H]^+^ *m/z*	Other Related *m/z* Peaks	Relative Abundance		Putative Identity	Ref.
					T24/T_N_	T26/T_N_		
1	0.5	175.0		115 (M-C_2_H_4_O_2_) 215 (M+K) 199 (M+Na)	4.46	2.62	Ascorbic acid ^b^	
2	4.4	193.0	195.0	177 (M-OH)	1.59	0.99	Ferulic acid ^b^	
3	4.9	787.2		655.1 (M-apiose-H) 331 (patuletin -H)	3.00	1.14	Patuletin-3-glucosyl-(1→6) [apiosyl(1→2)] glucoside	[32,33]
4	5.2	639.2	641.2	611.2 (M- 2H_2_O) 663.2 (M+Na)	4.24	2.78	Flavonol diglycoside	[34]
5	5.5		481.3	463.3 (M-OH) 445.3 (M-2OH) 427.3 (M-3OH)	2.48	0.89	20-hydroxyecdysone	[35]
6	6	429.3	431.3	453.3 (M+Na)	2.34	1.32	α-tocopherol	[36]
7	6.1	535.4	537.4		2.85	0.87	Jaceidin glucuronide	[33]
8	6.2	521.1	523.1		2.38	1.40	Spinatoside	[32,33]
9	6.4	519.1	521.1	309 (M-glucuronide)	2.77	1.15	5,3′,4′-Trihydroxy-3-methoxy-6:7-methylendioxyflavone-4′-glucuronide	[32,33]
10	6.5	533.1	535.1	557.1 (M+Na) 505.1 (M-MeO)	2.59	0.94	5,4′-dihydroxy-3,3′-dimethoxy-6:7-methylendioxyflavone-4′-glucuronide	[32,33]

^a^ Principal identified peaks and relative abundance referred to the control and hypothesis about chemical identity. T24 and T26: sampling times after 24 h of hypoxia (T24) and further 2 h of re-oxygenation (T26). TN relative normoxic control. ^b^ Compared with the standard compound.

**Table 2:** Trypan blue exclusion cell viability assay.

	Juice Concentration (%)	Mortality (%)
**C**	0	3
*Normoxic Condition*		
**T24_N_**	10	1
	25	2
	50	39
**T26_N_**	10	11
	25	13
	50	17
*Stress Condition*		
**T24_H_**	10	3
	25	2
	50	71
**T26_H_**	10	28
	25	15
	50	17

^2^ Mortality (%) induced by juice of spinach plants, sampled at different times of oxygen deficiency, grown under normoxic (N, control plants) or hypoxic (H, stressed plants) conditions, tested at three different concentrations (10%, 25%, and 50%) on HT29 cells. C: untreated cells. T24: cells treated with spinach juice from normoxic/hypoxic plants. T26: cells treated with spinach juice from normoxic/hypoxic plants 2 h after the re-oxygenation.

## Supplementary Materials

The following are available online at http://www.mdpi.com/2218-273X/9/2/53/s1, Table S1: Monitoring of changes in O^2^ levels in the nutrient solution. Figure S1: LC-MS profile of the extract. Figure S2: Negative mode ESI-MS spectra. Figure S3: Comet Assay.
