# Supplementary material for: Oxygen Availability during Growth Modulates the Phytochemical Profile and the Chemo-Protective Properties of Spinach Juice
Source: Biomolecules. 2019 Feb 4;9(2):53. doi: 10.3390/biom9020053 (PMC6406831; doi:10.3390/biom9020053)

biomolecules

Article

Oxygen Availability During Growth Modulates the Phytochemical Profile and the Chemoprotective Properties of Spinach Juice

Francesco Milano [1], Francesca Mussi [1,2], Silvia Fornaciari [2], Meltem Altunoz [2], Luca Forti [2], Laura Arru [2,*] and Annamaria Buschini [1,3]

[1] Dipartimento di Scienze Chimiche, della Vita e della Sostenibilità Ambientale, Università degli Studi di Parma, Parco Area delle Scienze 11/A, 43124 Parma, IT; francesco.milano@gmail.com (F.M.); francesca.mussi@unipr.it (F.M.); annamaria.buschini@unipr.it (A.B.)

[2] Dipartimento di Scienze della Vita, Università degli Studi di Modena e Reggio Emilia, via Amendola 2, 42122 Reggio Emilia, IT; fornaciari04@gmail.com (S.F.); meltem.altunoz@unimore.it (M.A.) luca.forti@unimore.it (L.F.); laura.arru@unimore.it (L.A.)

[3] COMT (Centro di Oncologia Molecolare e Traslazionale), Università degli Studi di Parma, Parco Area delle Scienze 11/A, 43124 Parma, IT; annamaria.buschini@unipr.it (A.B.)

Supplementary materials

Table S1. Monitoring of changes in O_2 levels in the nutrient solution.

Changes in O_2 levels in the nutrient solution used for hydroponic spinach growth after during normoxia, induction of hypoxia and subsequent reoxygenation. Values refer to tanks with 10 L of nutrient solution and 44 plants per tank.

Day	time	air saturation (%)	O_2 (mg/l)	water temperature (°C)
		NORMOXIC TREATMENT		
**day 1**	9:30	81	7.1	21.5
	10:30	83	7,2	22.0
	11:30	84	7.2	22.9
	12:30	80	6.8	22.8
	13:30	79	6.6	23.3
	15:30	80	6.7	23.9
	17:30	81	6.7	24.5
	19:30	84	6.8	24.9
	21:30	83	6.8	24.8
**day 2**	11:30	86	7.1	23.5
	13:00	86	7.0	24.8
HYPOXIC TREATMENT				
**day 1**	9:30	87	7.4	21,9
	10:30	83	7.2	21.6
	11:30	84	7.3	22.0
	12:30	80	6.8	22.4
	13:30	68	5.8	23.1
	15:30	56	4.7	23.6
	17:30	46	3.8	24.3
	19:30	39	3.2	24,8
	21:30	34	2.8	24.6
**day 2**	11:30	12	1.0	23.8
	13:00	6	0.5	24.7
REOXYGENATION				
**day 2**	9:30	32	2.7	21.5
	10:00	63	5.5	22.0
	11:00	70	6.0	22.5
	13:00	74	6.2	23.0

Figure S1. LC-MS profile of the extract

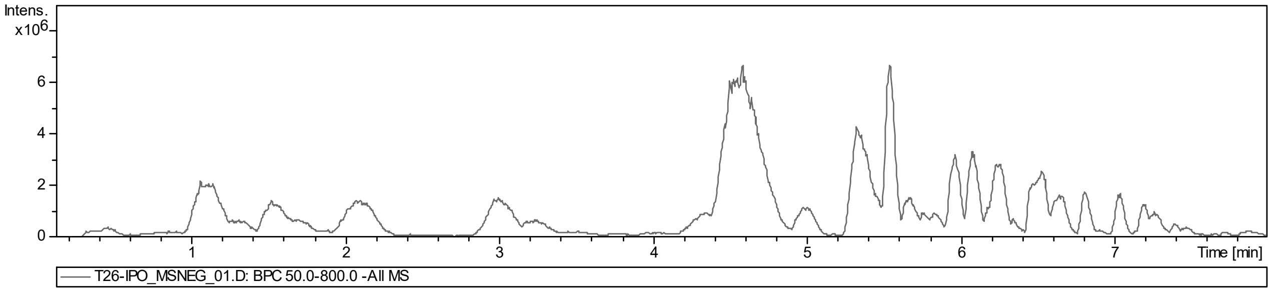

Figure S2. Negative mode ESI-MS spectra

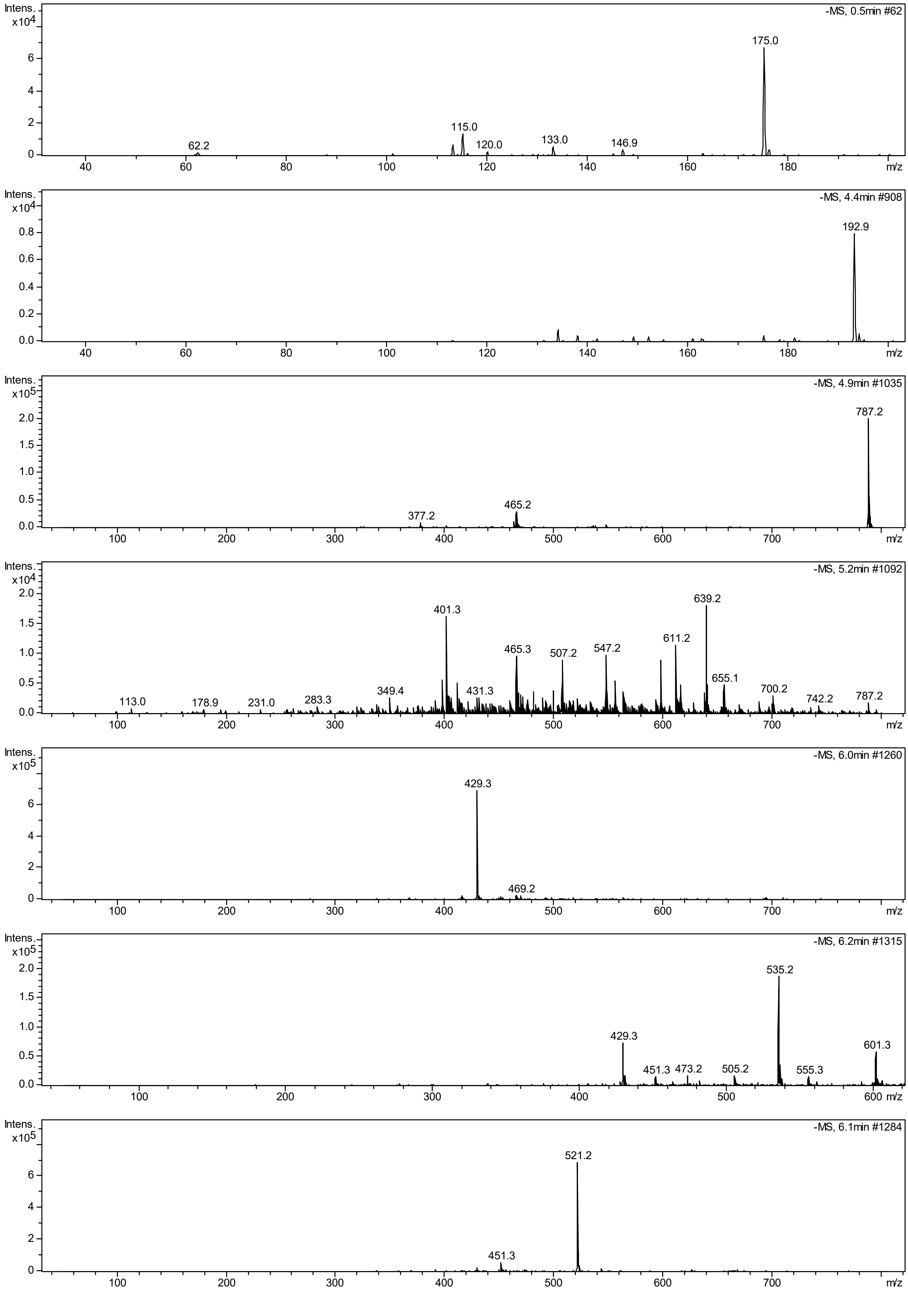

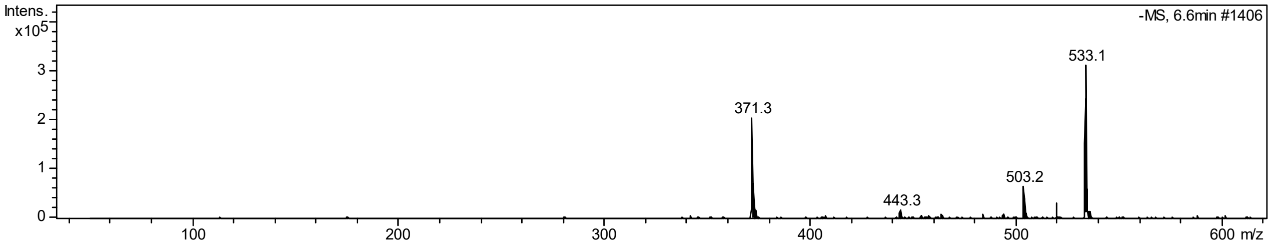

Intens.
x10^5
-MS, 6.6min #1406
533.1
371.3
503.2
443.3
100
200
300
400
500
600 m/z

Figure S3. Comet Assay

Images of HT-29 nuclei of cells treated with: A – untreated cells; B- H_2O_2 (100 μM, 5 min); C - normoxic juice (5%, 24h)+ H_2O_2 (100 μM, 5 min); D - hypoxic juice (5%, 24h) + H_2O_2 (100 μM, 5 min); E reoxigenated juice (5%, 24h hypoxia + 2h reoxigenation) + H_2O_2 (100 μM, 5 min).

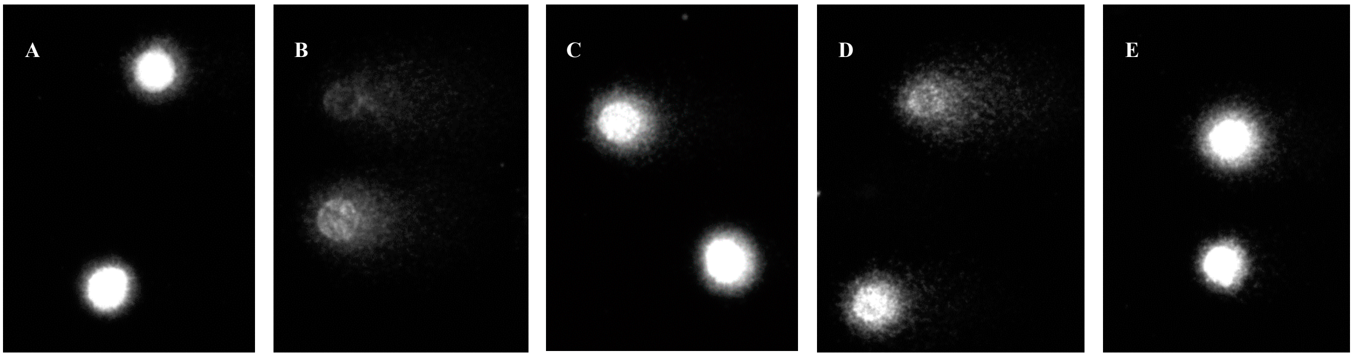

Supplement: Supplementary file 1 [file biomolecules-09-00053-s001.pdf]
